# Neurogenomics and the role of a large mutational target on rapid behavioral change

**DOI:** 10.1186/s13062-016-0162-1

**Published:** 2016-11-08

**Authors:** Craig E. Stanley, Rob J. Kulathinal

**Affiliations:** 1 Department of Biology, Temple University, Philadelphia, PA 19122 USA; 2 Institute of Genomics and Evolutionary Medicine, Temple University, Philadelphia, PA 19122 USA

**Keywords:** Behavior, Genomic architecture, Long genes, Neurodevelopmental disease, Neurogenome, Rapid evolution, Sexual isolation

## Abstract

**Background:**

Behavior, while complex and dynamic, is among the most diverse, derived, and rapidly evolving traits in animals. The highly labile nature of heritable behavioral change is observed in such evolutionary phenomena as the emergence of converged behaviors in domesticated animals, the rapid evolution of preferences, and the routine development of ethological isolation between diverging populations and species. In fact, it is believed that nervous system development and its potential to evolve a seemingly infinite array of behavioral innovations played a major role in the successful diversification of metazoans, including our own human lineage. However, unlike other rapidly evolving functional systems such as sperm-egg interactions and immune defense, the genetic basis of rapid behavioral change remains elusive.

**Presentation of the hypothesis:**

Here we propose that the rapid divergence and widespread novelty of innate and adaptive behavior is primarily a function of its genomic architecture. Specifically, we hypothesize that the broad diversity of behavioral phenotypes present at micro- and macroevolutionary scales is promoted by a disproportionately large mutational target of neurogenic genes. We present evidence that these large neuro-behavioral targets are significant and ubiquitous in animal genomes and suggest that behavior’s novelty and rapid emergence are driven by a number of factors including more selection on a larger pool of variants, a greater role of phenotypic plasticity, and/or unique molecular features present in large genes. We briefly discuss the origins of these large neurogenic genes, as they relate to the remarkable diversity of metazoan behaviors, and highlight key consequences on both behavioral traits and neurogenic disease across, respectively, evolutionary and ontogenetic time scales.

**Testing the hypothesis:**

Current approaches to studying the genetic mechanisms underlying rapid phenotypic change primarily focus on identifying signatures of Darwinian selection in protein-coding regions. In contrast, the large mutational target hypothesis places genomic architecture and a larger allelic pool at the forefront of rapid evolutionary change, particularly in genetic systems that are polygenic and regulatory in nature. Genomic data from brain and neural tissues in mammals as well as a preliminary survey of neurogenic genes from comparative genomic data support this hypothesis while rejecting both positive and relaxed selection on proteins or higher mutation rates. In mammals and invertebrates, neurogenic genes harbor larger protein-coding regions and possess a richer regulatory repertoire of miRNA targets and transcription factor binding sites. Overall, neurogenic genes cover a disproportionately large genomic fraction, providing a sizeable substrate for evolutionary, genetic, and molecular mechanisms to act upon. Readily available comparative and functional genomic data provide unexplored opportunities to test whether a distinct neurogenomic architecture can promote rapid behavioral change via several mechanisms unique to large genes, and which components of this large footprint are uniquely metazoan.

**Implications of the hypothesis:**

The large mutational target hypothesis highlights the eminent roles of mutation and functional genomic architecture in generating rapid developmental and evolutionary change. It has broad implications on our understanding of the genetics of complex adaptive traits such as behavior by focusing on the importance of mutational input, from SNPs to alternative transcripts to transposable elements, on driving evolutionary rates of functional systems. Such functional divergence has important implications in promoting behavioral isolation across short- and long-term timescales. Due to genome-scaled polygenic adaptation, the large target effect also contributes to our inability to identify adapted behavioral candidate genes. The presence of large neurogenic genes, particularly in the mammalian brain and other neural tissues, further offers emerging insight into the etiology of neurodevelopmental and neurodegenerative diseases. The well-known correlation between neurological spectrum disorders in children and paternal age may simply be a direct result of aging fathers accumulating mutations across these large neurodevelopmental genes. The large mutational target hypothesis can also explain the rapid evolution of other functional systems covering a large genomic fraction such as male fertility and its preferential association with hybrid male sterility among closely related taxa. Overall, a focus on mutational potential may increase our power in understanding the genetic basis of complex phenotypes such as behavior while filling a general gap in understanding their evolution.

**Electronic supplementary material:**

The online version of this article (doi:10.1186/s13062-016-0162-1) contains supplementary material, which is available to authorized users.

## Background

The diversity of behavioral phenotypes in the animal kingdom range from the subtle and cryptic to the extraordinarily bizarre. Behaviors such as territoriality, foraging, mating, predation avoidance, parental care, and communication generally evolve as newly derived traits to optimize the overall fitness of individuals with their surrounding environment and with each other. Such adapted behavioral traits are heritable and can evolve extremely quickly across relatively few generations (e.g., [1, 2]). However, it is precisely this rapid evolution that makes behavior difficult to study using a comparative approach. Behavioral traits, when compared to morphological, physiological, and life history traits, show relatively little phylogenetic signal [3–5] owing to behavior’s highly derived and labile nature. In addition, our understanding of the genetic basis of behavior is limited due to polygenic inheritance, our modest understanding of the complex interplay between development and physiology on neural circuitries, and plasticity in gene expression and cellular modeling in the face of variable environments [6, 7].

**Fig. 1:**
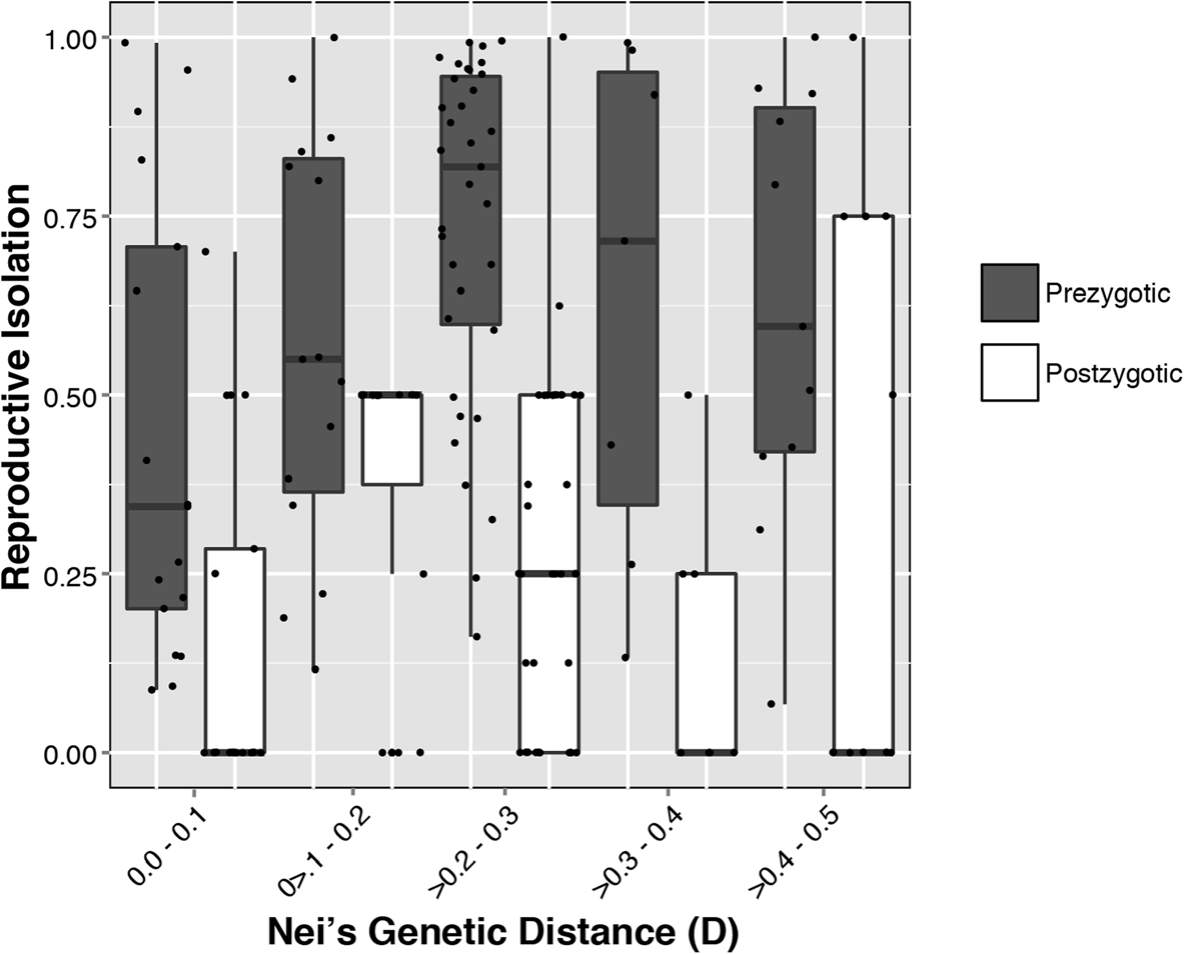
Prezygotic (sexual) isolation evolves faster than postzygotic isolation. Re-drawing of data from Coyne and Orr (1997) contrasting premating (behavior) and postzygotic (sterility/inviability) rates of reproductive isolation across 86 Drosophila species pairs over a range of divergence times. Nei’s Genetic Distance (D) is used as a proxy for divergence. Only species pairs with both pre- and postzygotic isolation estimates were used

While the species-specific nature of behavior makes it difficult to compare how behavior evolves relative to other functional classes, meta-analyses on multiple species highlight their exceptional rate of change and importance in generating species diversity. In their classic study, Coyne and Orr [8] compare genetic distance against the degree of reproductive isolation using hundreds of hybridizable species pairs from Drosophila and found that behavioral (prezygotic) isolation evolves much faster than postzygotic (F_1_ sterility/inviability) isolation (Fig. 1). While Coyne and Orr [8] noted that rate differences disappear when sympatric species are ignored, this pattern suggests that behavioral differences can, and often, evolve first during species divergence. Similar results are found in birds [9], cichlids [10], and other fish [11], and point to the commonality of rapid behavioral shifts across the animal kingdom in promoting species diversity.

The importance of rapid behavioral change can also be seen on a much smaller evolutionary timescale. Domesticated behaviors such as tameness and docility evolve extremely rapidly, often in the span of a breeder’s lifetime (e.g., [1, 12], [13]). Many livestock and pets have been bred for desired traits demonstrating the rapidity of adaptive change across heritable behavioral characters. Furthermore, experimental populations also demonstrate how selection can quickly change behavioral preferences within single digit generation times [2]. Thus, both domestication and experimental population studies demonstrate the ease in which selection can rapidly transform a population’s behavioral interactions with its immediate environment, even despite diminished levels of genetic variation [14].

As seen in studies of artificial selection, from domesticated organisms to experimental evolution, selection can quickly drive behavioral change. So how does behavior evolve so rapidly and in a species-specific fashion, and what is its molecular signature? Rapidly evolving genetic systems typically show strong signatures of divergence at the genic level pointing to an underlying molecular mechanism. For example, both immunity and reproduction are among the most diverged functional classes and encode genes that are similarly highly diverged [15–17]. These genes often harbor higher rates of amino acid replacement in their protein-coding regions, indicative of relaxed or positive selection. Therefore, one may similarly, and naively, expect higher mean divergence among neurogenic genes (i.e., higher K_a_/K_s_ in genes expressed in neural tissues such as brain) compared to genes from other more conserved genetic systems. However, neurogenic genes are generally known to be well conserved [18–20]. In addition, molecular signals of adaptation in neurogenic proteins may be too subtle to identify due to the regulatory, polygenic, and epistatic nature of the behavioral code. It’s telling that despite decades of studying sexual isolation in many different animal taxa, we still have not identified behavioral genes that drive reproductive isolation.

### Presentation of the hypothesis

While fast evolving genes generally encode rapidly evolving genetic systems, the converse is not always true. Our case in point: rapidly evolving behaviors are usually encoded by relatively conserved neurogenic genes. To explain this discordance, we suggest that the rapid emergence of behavioral novelty throughout the animal kingdom is a consequence of the disproportionately large size of its neurogenic targets. Large neuro-mutational targets can then accelerate the evolution of behavioral phenotypes in metazoan taxa with nervous systems by providing large functional structural and regulatory substrates for evolutionary, genetic, and molecular mechanisms to act upon. Thus, instead of a direct correlation of evolutionary rates between behavioral phenotypes and their underlying genes, we expect neurogenic genes to cover a large functional fraction of the metazoan genome.

To explain how a large mutational target can effect rapid behavioral change, we offer several potential mechanisms. First, larger genomic targets may simply provide a proportionally larger number of variants for selection and/or drift to act upon. This hypothesis places mutational input [21] at the forefront of rapid behavioral change. It has also been shown that larger genes tend to produce more transcripts through alternative splicing [22] resulting in a greater potential to generate phenotypic novelty [23]. Thus, a richer variant pool promotes *evolutionary* processes such as selection and drift, with recombination providing an accelerated path to ascend fitness peaks [24]. Second, a larger allelic pool may increase phenotypic plasticity and associated evolutionary diversification [25]. This *genetic* explanation places a dynamic signaling environment, full of biotic and abiotic cues, as driving adaptive behavioral response via heritable neuroplasticity [26]. Third, large genes have recently been found to exhibit unique *molecular* properties that may contribute to the variation in expression of neuro-behavioral phenotypes [27]. Recent work has shown that double-strand breaks [28], transposable elements [29] and *de novo* mutations in transcriptional repressors that affect topoisomerase activity [30] have a greater effect on large neural genes and have been recently implicated in a number of neurodevelopmental disorders.

Lastly, we predict that neurogenic genes will cover a significant fraction of the genomes of taxa with nervous systems and an ability to respond to complex stimuli. The complexity of behavioral phenotypes in a given taxa is expected to correlate with the size of the neurogenome across broad phylogenetic distances from ctenophores to bilaterans [31]. Neurogenetic genes provide the basis for complex metazoan behavior to evolve including the ability to propagate action potentials, transmit and receive neurotransmitters, and produce and maintain long axonal processes. Collectively, these genes permit animals to integrate multiple signals and generate appropriate responses by communicating intercellularly over large distances nearly instantly.

### Testing the hypothesis

#### Neuro-behavioral genes are larger, but more conserved

**Fig. 2:**
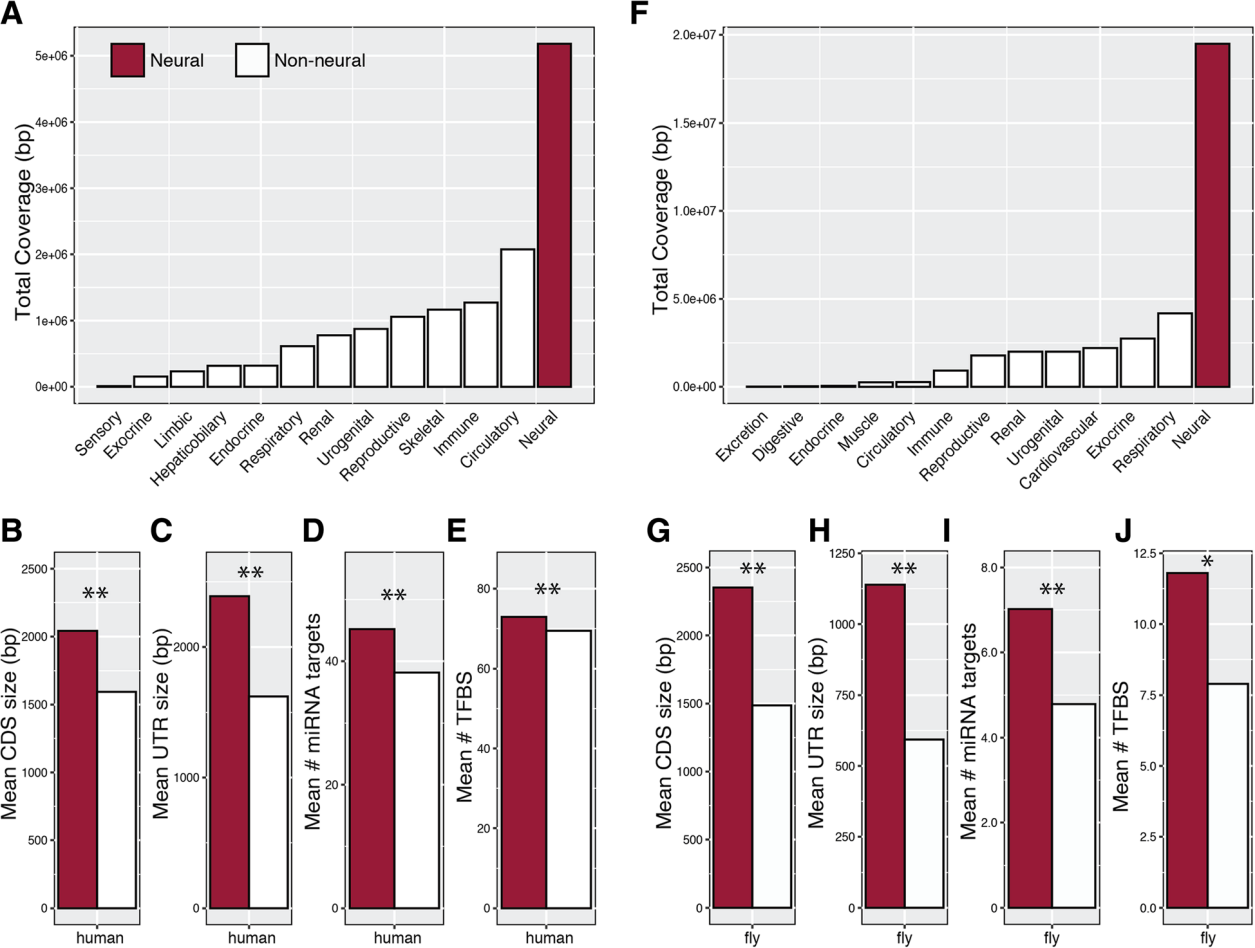
Disproportionately large neurogenetic targets in humans and flies. Total genomic coverage for the ontological class “neurogenetic” in (**a–e**) *Homo sapiens* and (**f–j**) *Drosophila melanogaster*. Average per gene size of coding region, CDS (**b, g**), untranslated regions, UTRs (**c, h**), number of microRNA targets, miRNA (**d, i**), and the number of transcription factor binding sites, TFBS (**e, j**). Asterisks above each comparison indicate significant differences between neural and non-neural gene sets using a Wilcoxon rank sum test (*P < 0.05, **P < 0.01)

Recent work provides evidence that mammalian brains and neuronal tissue express genes that are longer on average than other tissues [30]. A similar study also found that transcriptomes from various neuronal subtypes are significantly biased towards longer transcripts when compared to non-neuronal cell types [32]. To test whether other animal taxa exhibiting complex behavior possess the same pattern, we first compared different functional ontologies using expertly curated annotations from humans and flies, with each gene separated into coding and non-coding regions. In each of the reference genomes, neurogenic genes encode a disproportionately larger genomic target when compared to other hierarchically-similar, systems-based ontologies (Fig. 2a and f). In humans, neurogenic genes comprise approximately five percent of genes in humans yet cover approximately a tenth of the total genic space. Specifically, we find that neurogenic genes contain significantly larger CDS (Fig. 2b and g) and UTR (Fig. 2c and h) regions along with an increased regulatory repertoire harboring a greater number of microRNA targets (miRNA; Wilcoxon *P* < 0.05; Fig. 2d and i) ﻿﻿and transcription factor binding sites (TFBS; Wilcoxon *P < 0.01;* Fig. 2e and j).

**Fig. 3:**
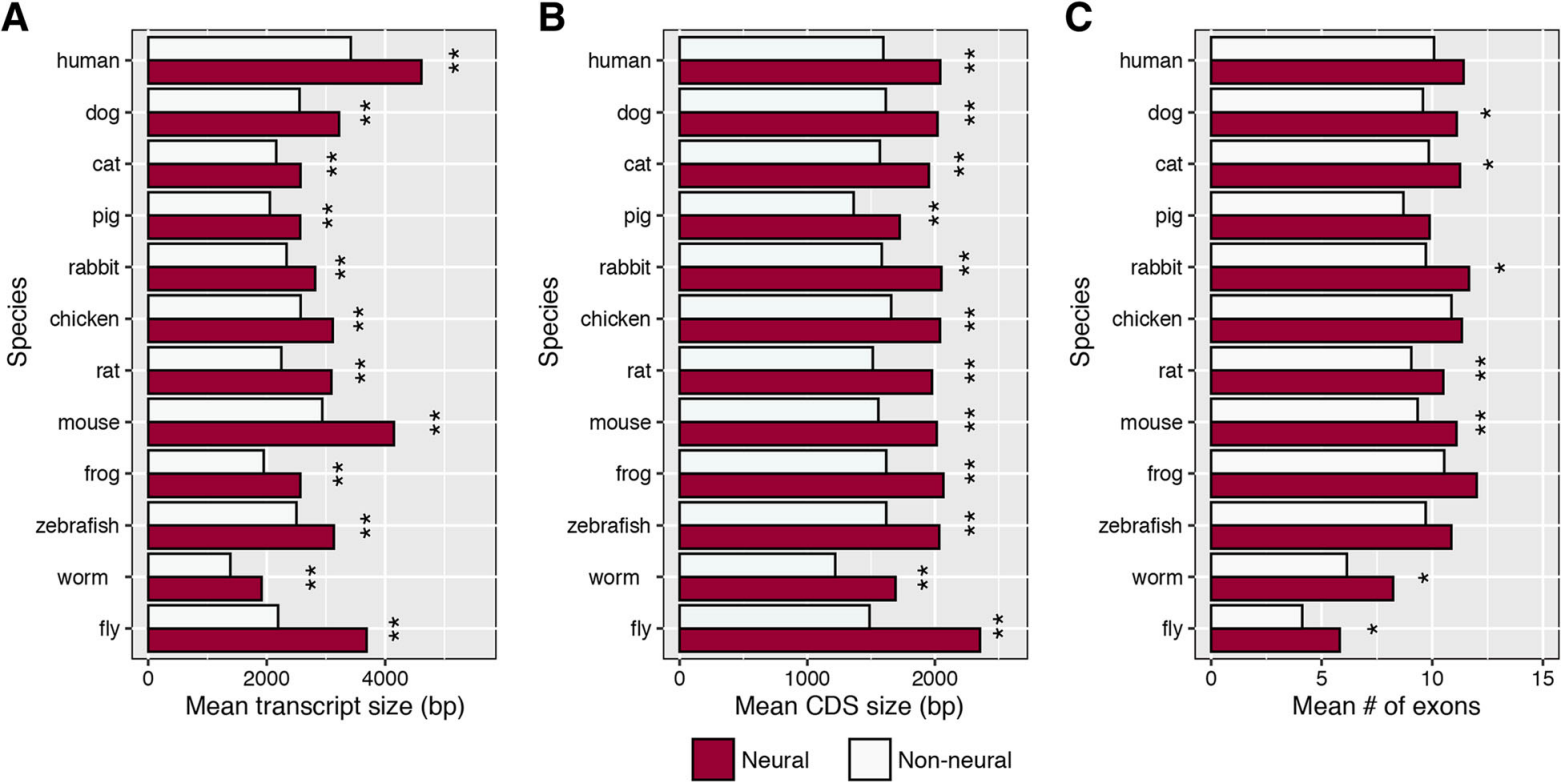
Large metazoan neurogenome. Total base pair coverage of neurogenetic vs non-neurogenetic genes across a dozen metazoans. **a** Mean transcript sequence length, (**b**) Mean coding DNA sequence length, CDS, (**c**) Mean number of exons per gene. Analyses based on human gene ontology annotations. Asterisks next to individual species comparisons indicate significant differences between neural and non-neural gene sets using a Wilcoxon rank sum test (*P < 0.05, ** P < 0.01)

Do similar patterns exist in other metazoan groups? Using additional taxa, each with annotated genomic data, we compared a similar set of characteristics among neurogenic genes. Again, across all taxa examined including ctenophores (Additional file 1: Figure S1), neurogenic genes were longer in both transcript and CDS length with some species possessing a greater number of exons, potentially suggesting a greater transcript diversity due to alternative splicing (Fig. 3).

**Fig. 4:**
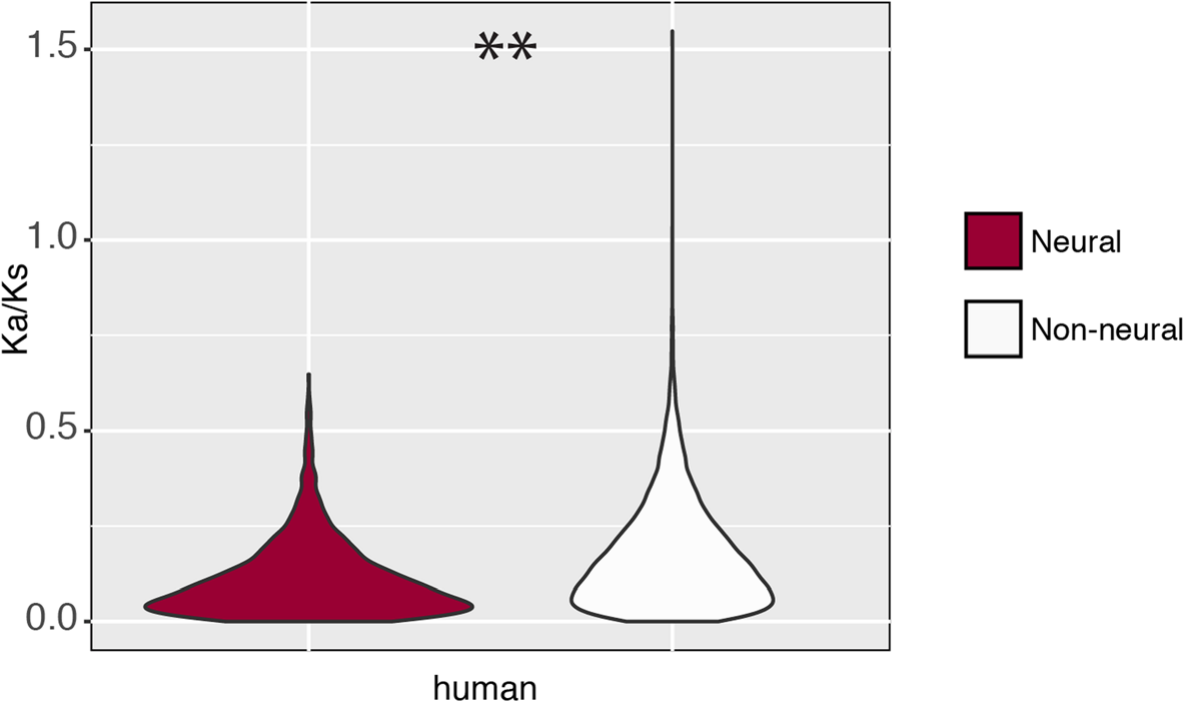
Neurogenetic genes are more conserved. Violin plots based on estimates of selection (K_a_/K_s_) for neural vs non-neural genes between human and mouse. Asterisks above each comparison indicate significant differences using a Wilcoxon rank sum test (**P < 0.01)

While the neurogenome has a large footprint, its encoded proteins do not exhibit higher divergence rates, in terms of K_a_/K_s_, between human and murine genomes (Fig. 4) indicating that neither positive Darwinian selection nor relaxed selection correlates with the rapid divergence of behavioral phenotypes. On the contrary, amino acids appear to be more conserved (Wilcoxon *P < 0.001*, Fig. 4), as reported previously [18].

#### Mechanisms of rapid behavioral evolution

While a significantly larger neurogenomic target is observed across animal taxa, its role in effecting rapid behavioral change remains unknown. The recent ease and low cost of sequencing should help differentiate among a variety of mechanisms. One hypothesis is that a larger neurogenomic target simply increases the number of functional sites available to quickly transform behavioral phenotypes via drift and selection. Surveying GWAS SNPs with a significant effect size on behavioral phenotypes or disease (e.g., [33]) will inform us about the nature and relative frequency of such mutations in regulatory vs. structural regions of the neurogenome. Transcriptomes from neural tissues and cell types can also be sequenced to identify candidate *de novo* transcripts to eventually be assayed for behavioral effects. Whether the same variant has a different phenotype in other populations with divergent environmental cues may also help us understand the role of allelic variation on behavioral plasticity. In this case, the magnitude of adaptive behavioral plasticity is proportional to the number of allelic variants with variable heritable responses across changing environments [34]. Genetic models such as Drosophila, nematodes, and mouse, that can control both the genetic background and environmental conditions as well as accurately quantify behaviors, will be an important tool to address the extent of adaptive behavioral plasticity [35]. Furthermore, we note that large genes, due to their shear size, may also harbor a greater number of mobile DNA elements and double-strand breaks that result in phenotypic change. For example, transposable elements have recently been found to actively retrotranspose during neurogenesis, providing a dynamic regulatory mechanism to effect rapid behavioral responses [29].

On the other hand, there is mounting evidence that such disruptive events are inherently biased towards large genes. In a recent study, recurrent double-strand breaks (DSB) were found to be enriched in long genes, even when normalized for gene size, demonstrating that longer genes are more susceptible to DSB-induced genomic instability [28]. These somatic changes are believed to help generate greater neuronal diversity via genomic mosaicism [29]. Alternatively, a few genes may control the downstream expression of large neurogenic genes due to their unique transcriptional properties. Recently, a higher rate of neurodevelopmental disorders was found to be caused by topoisomerase disfunction that generally targets large genes via epigenomic mechanisms [36]. In particular, *de novo* mutations of methyl-CpG-binding protein 2 (MECP2), a transcriptional repressor of large genes [37], causes the X-linked neurodevelopmental disorder, Rett’s syndrome. Thus, small changes in a neurodevelopmental “control” gene can single-handedly transform the expression of a broad cast of large neurogenic genes. The use of genome editing tools to change/delete specific sites or gene regions will certainly assist us in understanding the genetic etiology of behavior.

#### On the origins of behavioral diversity

The large mutational target hypothesis predicts that the range of behavioral phenotypes expressed in an organism is proportional to the size of its total underlying neurogenic code. Thus, a comparative genomics analysis on taxa with differing levels of behavioral complexity could be applied across diverse metazoan lineages. Fortunately, large and conserved neurogenic genes present ideal markers to identify distant orthologs needed to study the evolutionary origins of behavioral novelty. In addition, comparative *de novo* transcriptomic analyses can quickly provide new insight, even in taxa without an assembled genome. The large mutational target hypothesis also predicts a greater birth rate of neurogenic genes in species that have evolved new complex behavioral adaptations such as gene family expansions of neuronally-expressed protocadherins and C2H2 zinc-finger transcription factors in cephalopods [38]. In addition, the application of a genomic phylostratigraphical approach [39], based on well-studied neurogenic genes in mammals and insects, may allow researchers to time the origin of behavioral innovations (a task nearly impossible to perform with behavior using the fossil record) by mapping neurogenic genes to ancestral nodes. Finally, applying functional assays on a number of these ancient large genes—the early progenitors of today’s neurotransmitter receptors, axon guidance factors, signaling molecules, and other neural-related functions—will tell us much about the evolution of ancient nervous system function and the origins of complex behavior in early metazoans.

### Implications of the hypothesis

Studying the genes that underlie genetic systems has emerged as a powerful framework to understand and infer rates of phenotypic change [15, 40]. Rates of evolutionary change at the molecular level often correlate with rates of phenotypic change providing insight into species-level processes that occurred in the past. For example, conserved phenotypes such as cellular growth and metabolism are typically encoded by slowly evolving genes and indicate the critical and ubiquitous importance of these essential genes for basic survival. On the other hand, rapidly evolving systems such as immunity and reproduction are encoded by some of the fastest evolving proteins suggesting their involvement in a dynamic arms races with multiple genes co-evolving under positive Darwinian selection. Subsequently, signatures of negative and positive selection in genomic sequences are often used to predict the extent of phenotypic change. However, among many genetic systems, the correlation between molecular and phenotypic evolutionary rates often fails due to the polygenic and regulatory nature of complex phenotypes, functional pleiotropy, and species-specificity that removes traits and genes from the comparative framework. Thus, a complementary framework is needed.

#### Mutation’s key role driving rapid behavioral change

The large-target effect places mutation at the forefront in generating phenotypic innovation. Mutation’s perceived role in driving evolutionary patterns has changed over the last century from a position of prominence [41, 42] to enabling selection [43]. This duality—the randomness of mutation coupled to deterministic selection—provides a dynamic in which adaptations are quickly generated and fixed, and fits contemporary views that organismal complexity is driven by selection on a genetic landscape originally populated by non-adapted processes [44, 45]. When an entire spectrum of mutations (e.g., point mutations, indels, isoforms, CNVs) and epistatic interactions are involved, linear increases in the mutational landscape suddenly become exponential with respect to the number of recombined haplotypes for selection or drift to act upon.

#### Structural vs. regulatory effects on behavioral change

Behavior exemplifies the disconnect between genotypic and phenotypic rates of change: relative to other functional gene classes, its underlying neurogenic genes do not show associated signals of rapid divergence. On the contrary, we find lower mean K_a_/K_s_ indicating greater functional constraints at the protein level, indicating that structural mutations may impose a greater effect on fitness per amino acid unit (Fig. 4). This general pattern of lower d K_a_/K_s_ has been previously observed in comparative studies of genes expressed in the brain [18–20]. Stronger purifying selection on the protein alternatively may suggest that rapid evolution is primarily driven in the regulatory region. Thus, the rapid rate of change and extraordinary diversity found in animal behaviors may ultimately be driven by the disproportionately large regulatory size of its underlying neurogenic genes. Behavior may exemplify this “evo-devo” view (e.g., [46]) by evolving predominantly through cis-regulatory but not structural protein change.

#### Neurogenomics and reproductive isolation

The large mutational target effect impacts how variation accumulates, is maintained, and becomes eventually utilized in a population [21, 47]. Large neurogenic genes and genetic systems play a key role in behavior influencing not only survival, but also mate choice and recognition. A large pool of behavioral mutations provides an abundance of additive genetic variation for sexual selection to choose from [43, 48] and should correlate with rates of behavioral change. These behavioral variants may then play a key role in forming new species [49]. For example, adaptive radiations such as anole lizards [50], Hawaiian Drosophila [51], and cichlids [52] highlight rapid behavioral change in habitat and mate preferences [53].

Recent studies on the genomics of speciation in closely related species and divergent populations identified candidate genes involved in behavioral preferences that enable species to better adapt to their immediate abiotic and biotic surroundings (e.g., [54, 55]). Large differences in behavioral phenotypes are especially observed among so-called sibling species indicative of behavior’s dynamic nature. In fact, sexual isolation is thought to be a common driver of speciation [56] with sexually dimorphic behaviors such as preference and aggression changing rapidly within species as a result of sexual selection [57], and include models of accelerated runaway selection [58], sexual conflict [59], and mating discrimination between species due to reinforcing mechanisms [60]. Thus, behavior’s uniquely large genomic architecture presents a common mechanism in driving species divergence in animal taxa.

#### Rapid behavioral change in domesticated animals

The availability of a large variant pool provides a substrate for rapid phenotypic change over short periods of time. The large-target effect can explain similarities in domesticated phenotypes, thus, offering an alternative genomic mechanism for the behavioral component of the “domestication syndrome” and the propensity for “tameness” in domesticates [61]. Darwin’s introductory chapter in Origins [62] highlights the rapidity of artificial selection in domestication, providing some of the most engaging examples of evolution in action. In recent years, numerous genomic studies have identified neurogenic genes involved in domestication [14, 63–65]. Additionally, Wilkins et al. [61] has implicated the potential role of neural crest cells in domestic species, focusing on common morphological phenotypes seen across domestic phenotypes. We suggest that these neural crest genes may provide a disproportionately large genomic target for rapid change to occur.

#### Polygenic adaptation and behavior

Many, if not most, species-specific behaviors are thought to be result of adaptive processes. The large target hypothesis places polygenic adaptation in the spotlight and highlights the need to develop more robust tests of selection by modeling fluctuating selection [66] or using systems-based approaches [67] to test adaptive hypotheses. The failure to identify adapted behavioral candidate genes is likely due to our inability to detect soft selective sweeps in this genome-scaled form of polygenic adaptation [68] with selective responses based on subtle allele frequency changes across many loci [69, 70]. This multi-allelic, polygenic framework partners both standing genetic variation and *de novo* mutations in promoting behavioral novelty. The polygenic nature of behavior also reduces the chances that SNPs and genes involved in neurodevelopmental disorders are discovered. The presence of large neurogenic targets makes it more difficult to elucidate the genetic basis of neurodevelopment as a disproportionate number of rare and subtle variants in populations greatly reduces the power of GWAS, resulting in a large missing heritability component in many neurological disorders [71].

#### Large-target effect and the commonality of complex disease

The large mutational target effect may also play a role on an ontogenetic time scale, i.e., within the lifetime of an organism. For example, the higher incidence of *de novo* diseases encoded by large genes such as cystic fibrosis may result from their larger mutational target. Veltman and Brunner [72] previously proposed that the frequency of disease is directly proportional to the mutational target size of its underlying genes, and suggest that the large number of genes involved in brain developmental and function may determine the high frequency of neurodevelopmental disorders in humans.

We further suggest that the well-known correlation between neurogenic spectrum disorders in children and paternal age [73–75] is a direct result of a large neuro-mutational sink. While female gamete production ceases at the onset of puberty, male gamete production continues throughout their lifetime allowing mutations to accumulate in germ-line cells. Since DNA damage in sperm correlates with age [76], males continually acquire mutations. Thus, the large mutational target effect may explain why neurodegenerative disorders have a higher probability of occurrence in children with older fathers, i.e., a paternal age effect (Table 1), in addition to manifesting an overall higher variance in phenotypic expression.

**Table 1:** Neurodevelopmental disorders associated with increased paternal age

Disease	Study
Autism	Hultman et al. 2010; Alter et al. 2011; Frans et al. 2013; Alter and Nesbit 2014
Schizophrenia	Svensson et al. 2012; Jaffe et al. 2013; Pederson et al. 2013
Dyslexia	Jayaskera and Street 1978; Saha et al. 2009
Rett Syndrome	Goriely et al. 2013
Neurofibromatosis	Jadayel et al. 1990; Kluwe et al. 2000; Conti et al. 2013

### Large-target effect in other functional systems

While the large mutational target effect provides a genomic explanation for the predominance of behavioral divergence in metazoan populations and species, this systems-based framework can also be applied to any over-represented functional system. In general, random mutations on functional classes of genes that cover a sizeable fraction of the genome—whether the result of larger genes, more genes, or both—will lead to these functional classes evolving faster with potentially important evolutionary consequences. For example, rodents possess nearly double the number of olfactory receptor genes, reflective of the tremendous range of smells that murines can adaptively respond to, compared to other mammals [77].

Another example is hybrid male sterility which, due to its commonality across taxa, has often been called a “rule” of speciation [78]. In Drosophila, nearly a quarter of the genome is transcribed in and near spermatogenic genes [79] and ~60 % of all genes are expressed in the testes [80]. Thus, as one of the largest functional targets in Drosophila, rapidly accumulating mutations (e.g., [81]) in male reproductive genes [79, 82] may promote the ubiquitous development of hybrid male sterility in fruit flies as an initial stage in speciation [8]. Thus, if we view a phenotype as the sum of its genetic parts, then the rate of phenotypic change becomes a function of its underlying genomic size.

## Conclusions

Behavior is among the most rapidly evolving and derived traits in the animal kingdom, yet we know very little about the genes, genetic architecture, and evolutionary forces that promote their rapid divergence [83] Here we present evidence that neurogenic genes are significantly longer, providing among the largest genomic coverage of any ontological class in metazoans. These data results direct our attention to genomic architecture and the underappreciated role of a large mutational target on effecting rapid phenotypic change. Such changes occur over developmental and evolutionary time scales and have direct and indirect consequences that include the rapid development of behavioral isolation between species, the commonality of domesticated behaviors, and paternal age effects on neurogenerative disorders. The large mutational target effect provides a much-needed mutational component to explaining system-wide phenomena across a diverse set of taxa and the evolvability of complex phenotypic traits [47, 84, 85]. This hypothesis places a greater emphasis on the neurogenomic landscape, regulatory mutations, and historical contingency (e.g., [86]) to offer new insight into how behavior can evolve so rapidly. It is intriguing to think that animal behavior, and its crucial importance to survival and reproduction, is largely driven by a genomic legacy of large yet conserved metazoan genes. With a greater comprehensive knowledge of genomes and their annotated genetic elements, this systems genomics framework provides many exciting new hypotheses to test.

## Methods

### Data sources

Descriptive data (total gene length, transcript length, UTR length, CDS length) for each taxa were downloaded from Ensembl v83. Orthologous relationships to humans (dog, cat, pig, rabbit, chicken, rat, mouse, frog, and zebrafish, and ctenophores) and fly (worm) were downloaded using Ensembl Biomart v83. Regulatory data for human miRNA targets and transcription factor binding sites (TFBS) were downloaded from microRNA.org and ChIPBase (rna.sysu.edu.cn/chipbase/). Drosophila specific tissue and size information (Fig. 2f–h) were identified using methods described in Stanley and Kulathinal [14]. miRNA and TFBS data for Drosophila were downloaded from RedFly (redfly.ccr.buffalo.edu/) and microRNA.org.

### Neural gene annotation

Functional gene annotations were performed using a gene ontology classification identified via Quick GO (http://www.ebi.ac.uk/QuickGO). Specifically, the ontology “nervous system development” was used to classify neural genes in humans. For comparisons across different tissues and ontologies, gene ontologies from the same GO hierarchical level were used.

### Statistical analyses

Since the distribution of gene lengths is non-Gaussian, Wilcoxon rank sum tests were used to compare differences between neural and non-neural genes. Comparisons between species rely both on the accuracy by which orthology is called and the quality of the genome annotation, therefore, comparisons between specific species sets were not conducted.

## Reviewers’ comments

Reviewers report 1: Itai Yanai, Institute for Computational Medicine, New York University, School of Medicine, USA and Vlad Grishkevich, Senior Research Scientist at ARIAD Pharmaceuticals, USA.

In their manuscript Stanley and Kulathinal put forward an interesting hypothesis that the fast evolution of the behavioral changes is primarily determined by the organization of neuronal genes in the genome. The authors invoke the reported observation that neuronal genes are longer and occupy a larger genomic fraction than other gene sets to argue that neural genes are disproportionately more likely targeted by mutations. Their hypothesis is that the accumulated mutations form the genetic basis for the fast evolution of behavioral phenotypes. Additional properties of large genes, like higher abundance of splice isoforms, may also contribute to this process. The authors examine a rather limited set of species from a phylogenetic perspective. In the abstract they write beautifully “In fact, it is believed that nervous system development and its potential to evolve a seemingly infinite array of behavioral innovations played a major role in the successful diversification of metazoans, including our own human lineage.” and in the main text that “The complexity of behavioral phenotypes in a given taxa is expected to correlate with the size of the neurogenome across broad phylogenetic distances from ctenophores to bilaterans [31].” To support these claims can the authors also include the ctenophores and other distant species in their analysis?

Authors’ response: *We thank Drs. Yanai and Grishkevich for their valuable comments and insights. We chose these multiple species with the goal to maximize phylogenetic coverage but without a substantial loss in the quality of their functional annotations. The challenge is twofold: 1) behavioral complexity is difficult to measure and 2) the most reliable neurogenic annotations are found in humans and flies, leaving non-model annotations focused on humans. With this caveat in mind, we followed the reviewers’ request to include other distant species and applied the same pipeline to ctenophores. We find similar statistical significant patterns of larger neural genes in these basal metazoans (see Additional file* *1* *: Figure S1). As better annotations and assemblies become available in the future, we hope that further analyses will be performed.*

We have a few concerns regarding the presented analyses. If neurogenic genes are defined as those expressed exclusively in the neural system how will they compare to other gene sets (blood, muscles, skin, etc.). Can the Fig. 2b–e and g–j analysis be repeated with muscle genes, for example, as the reference as opposed to the ‘non-neuronal’?

Authors’ response: *As described in the newly added methods section (see above), neurological genes for individual genes size components were identified using the ontology “nervous system development”. Muscle genes, and other functional gene sets requested by the reviewers pose a problem as they fall under multiple classes. From the tissue analysis in Drosophila (Fig.* *2* *), one can see that genes that are expressed in muscle compose a significantly smaller proportion of the genome.*

Does the non-neuronal gene set include housekeeping genes that are known to be short?

Authors’ response: *Small genes, such as housekeeping genes are commonly shared in many of the functional gene sets, and did not present an ascertainment bias.*

Moreover, gene length is not normally distributed, yet the authors use mean values to compare gene sets in the figures. For this reason, a whisker diagram, histogram or a scatter plot would be a better way to visualize gene lengths.

Authors’ response: *To better represent the underlying distribution of neurogenic gene size, we provide histograms across the broad range of assayed taxa (see Additional file* *2* *: Figure S2). Since, as the reviewers correctly point out, gene lengths are not normally distributed, we have used non-parametric statistics to compare gene sets throughout the manuscript, most with statistically significant differences (Figs.* *2* *and* *3* *), including when the median is compared.*

While this is a hypothesis paper, data are shown and therefore a methods section should be included so that the analyses are reproducible. For example, how the gene size and the genome fraction translated into gene numbers? How many neural genes are there? How was the set of neurogenetic genes curated?

Authors’ response: *We agree with the reviewers and have included a short Methods section. In* Additional file 3: Table S1*, we provide the number of neurogenic genes identified in each taxa.*

The authors should better explain the plausibility of their hypothesis. Specifically, it is generally accepted that behavioural traits are highly polygenic, with any single genetic change leading to small effects. If neural genes are more prone to accumulating mutations how does this explain fast behavioural changes?

Authors’ response: *As the reviewers note, behavioral traits are generally regarded to be highly polygenic. Accumulating mutations can play a role in rapid behavioral changes through a variety of ways, depending on such parameters as the effect size of a mutation on fitness, as well as effective population size. One can envision a scenario where a few small effect mutations provided a substantive increase in fitness and, thus, were effectively selected. A contrasting scenario involves the fixation of mildly deleterious neurogenic mutations due to background selection in species with small population sizes. In both cases, rapid behavioral evolution would simply result from selection on a greater number of neurogenic mutations rising to fixation. We further assert that with more mutations, there will be a greater number of novel epistatic interactions for selection to act upon. We make this clearer in the revision.*

It is important to ask how we might be able to identify specific alleles in the vast mutation space of the nervous system. The authors write quite interestingly that “Current approaches to studying the genetic mechanisms underlying rapid phenotypic change primarily focus on identifying signatures of Darwinian selection in protein-coding regions. In contrast, the large mutational target hypothesis places genomic architecture and a larger allelic pool at the forefront of rapid evolutionary change, particularly in genetic systems that are polygenic and regulatory in nature.” However, it remains unclear how the genomic architecture is to be explored given these nervous-system-specific parameters to identify the genetic basis of behaviour.

Authors’ response: *This post-ENCODE era holds the promise to provide researchers with a fine-scale molecular map of behavioral traits and we are excited that transcriptomic and epigenomic studies on various tissues and cell types are beginning to offer unprecedented resolution to identify the regions and genetic elements that contribute to behavior. A next step is to augment such surveys with comparative maps among populations and between closely related species. Such surveys will not only identify individual SNP differences, but can identify other mutational candidates such as indels, gene copy variation, and inversion/translocation breakpoints within regions that are expressed in neural tissues. Experimental validation using tools such as CRISPR can then be used to help validate the effects of behavioral candidates.*

The authors can take this opportunity to also note how the testing and excluding of alternative explanations: in other words, which data would allow one to reject this hypothesis?

Authors’ response: *This hypothesis may be tested on the genomes of lineages that show major differences in behavioral diversity. One obvious example is comparing the size of the neurogenome between metazoans vs non-metazoans. Unfortunately, our current knowledge of function in non-model organisms is based on GO annotations from models such as humans, thus, making these comparisons tautological and potentially misleading. We hope that this annotation bias will be improved in the near future.*

Finally, can the authors speculate on what keeps neurogenic genes long.

Authors’ response: *Long neurogenic genes comprise a variety of sub-neuro-classes including* neurogenesis, *synapse maturation, and regulation of nervous system development* *. Gene length appears conserved across metazoans, and their shear size, including intronic and UTR regions, may be maintained by purifying selection on regulatory domains.*

### Reviewers report 2: David Ardell, University of California Merced, USA

The authors present a conceptually appealing and straightforward hypothesis that the relatively large mutational target size of neurogenes may help explain the rapid divergence of behavior, including by means of polygenic adaptation. They cite and discuss evidence from many important preceding works and also present original statistical analyses confirming and extending the premise that neurogenes are relatively large in metazoa and that protein-coding neurogenes do not appear to have particularly elevated K_N_/K_S_ values.

Authors’ response: *We thank Dr. Ardell for his positive comments and critical review of our manuscript*.

Readers might benefit if the authors address the following additional points in their narrative:

1. The applicability of their hypothesis to innate vs adaptive behaviors, and consequently across the range of metazoa with behavioral repertoires and neural systems of diverse complexity. Although the abstract opens by naming innate behavior, the variety of examples discussed are not restricted to innate behaviors (nor should they be, necessarily).
  Authors’ response: *We agree that this hypothesis is not just restricted to innate behaviors and, accordingly, have included adaptive behaviors in the abstract.*
2. The roles of developmental and behavioral plasticity in both innate and adaptive behavior and their relevance to the hypothesis should perhaps be spelled out more explicitly. Even innate behavior, for example alarm calls, involve significant learning and development. Plasticity of neural substrates of behavior are discussed in the manuscript. But surely the multiple levels of plasticity in behavior should be taken into account when attempting to explain its rate of divergence relative to other traits? At least it should be mentioned that mutational target size cannot explain everything.
  Authors response: *We agree that a significant learning component involving neural plasticity is involved in many innate behaviors. A large mutational target simply promotes heritable change. We clarify this point in the paper. A recent paper in Nature Neuroscience (J.A. Erwin et al. 2016. L1-associated genomic regions are deleted in somatic cells of the healthy human brain. Nature Neuroscience doi:* 10.1038/nn.4388 *) demonstrates the potential usefulness of this hypothesis in understanding the learning process. In their work, the authors found LINE-1 retrotransposition events resulted in genetic deletions, promoting genomic mosaicism in the brain. Thus, large neurogenic genes provide a substantial genomic substrate for somatic neurogenic changes in progenitor and adult neural cells.*
3. Would some discussion of the Baldwin effect be appropriate in this context?
  Authors response: *The reviewer interestingly refers to the Baldwin effect which reflects an individual’s ability to learn novel behaviors. The Baldwin effect models the evolution of behavior* via *learning across generations. Our hypothesis presents a mechanism by which population genetic variation for a specific learning ability could evolve under mutation-selection.*
4. Not only neurogenes underlie behavioral traits genetically, but other genetic systems also contribute to behavior.
  Authors’ response: *Other genes, especially genes related to sensory categories and motor genes, certainly may contribute to behavior, therefore, increasing the diversity and overall coverage of neurogenes. These can also be considered as neurogenic genes, in the broad sense, due the interactive nature of behavior acting between other systems and the nervous system.*
5. Although the argument is clear regarding K_N_/K_S_, perhaps it should be mentioned that other approaches to measuring adaptation could be applied to this problem. Similarly, although polygenic adaptation is referenced, perhaps advances in this area or the need for them should be more explicitly called out in terms of testing the hypothesis.
  Authors’ response: *We agree and explicitly state the need to further develop polygenic tests of selection.*
6. For the analyses presented, the sources of data, i.e. annotations of transcription factor binding sites, microRNA targets, methods of statistical analysis should all be documented.
  Authors’ response: *The previous reviewers also expressed the need for this additional section, and we have added the appropriate documentation (see comment to Reviewer #1).*
7. Finally, although I am asking for a variety of points to be addressed, I do think that the manuscript as a whole would benefit from some tightening and shortening.
  Authors’ response: *In our latest version, we have done our best to tighten up the manuscript.*

Minor suggestions: 1. Page 5: “genetic basis of behavior is limited due to *their*” 2. Page 5: “ubiquitousness” 3. throughout: I find “neurogenic genes” to be redundant 4. Page 7: I think the authors mean that the “rate of fixation of mutations is proportional to overall target size.” 5. Page 8: “recent work reveal” 6. Page 8: “provide the basis for complex metazoan behavior” — should be “traits”? 7. Page 9. A non-significant result is reported with a significant Wilcoxon value, in reference to Fig. 4 8. Page 10: “there’s” “should be” there is“ 9. Page 10: The large mutational target *hypothesis* predicts” 10. Page 13: “A large pool of behavioral mutations …” ending with citations. Do these citations provide evidence for the claim, or is the claim itself being cited? 11. Page 15:” transcribed by spermatogenic genes” — is this what was meant? I found this phrasing confusing.

Authors response: *We thank Dr. Ardell for each of these detailed comments and suggestions and have made the appropriate changes in the manuscript.*

## Additional files

Additional file 1: Figure S1. Neural vs. non-neural gene length in ctenophores. (DOC 76 kb)
Additional file 2: Figure S2. Frequency distributions of gene lengths for neurogenic genes, across multiple taxa. (DOC 315 kb)
Additional file 3: Table S1. Number of neurogenic genes identified across taxa. (DOC 28 kb)

## Abbreviations

CDS: Protein-coding region sequence
CNV: Copy number variation
CpG: 5’-cytosine-phosphate-guanine-3’ sites
DSB: Double-strand break
GWAS: Genome-wide association study
K_a_/K_s_: Ratio of nonsynonymous substitutions per nonsynonymous site (K_a_) to the number of synonymous substitutions per synonymous site (K_s_), in the coding region of a gene
miRNA: microRNA
SNP: Single nucleotide polymorphism
TFBS: Transcription factor binding sites
UTR: Untranslated regions (5’ and 3’) that straddle the coding region of a gene on a strand of mRNA
