# Supplementary figures and images for: Neurogenomics and the role of a large mutational target on rapid behavioral change
Source: Biol Direct. 2016 Nov 8;11:60. doi: 10.1186/s13062-016-0162-1 (PMC5101817; doi:10.1186/s13062-016-0162-1)

Supplementary Figure 1. Neural vs. non-neural gene length in ctenophores.


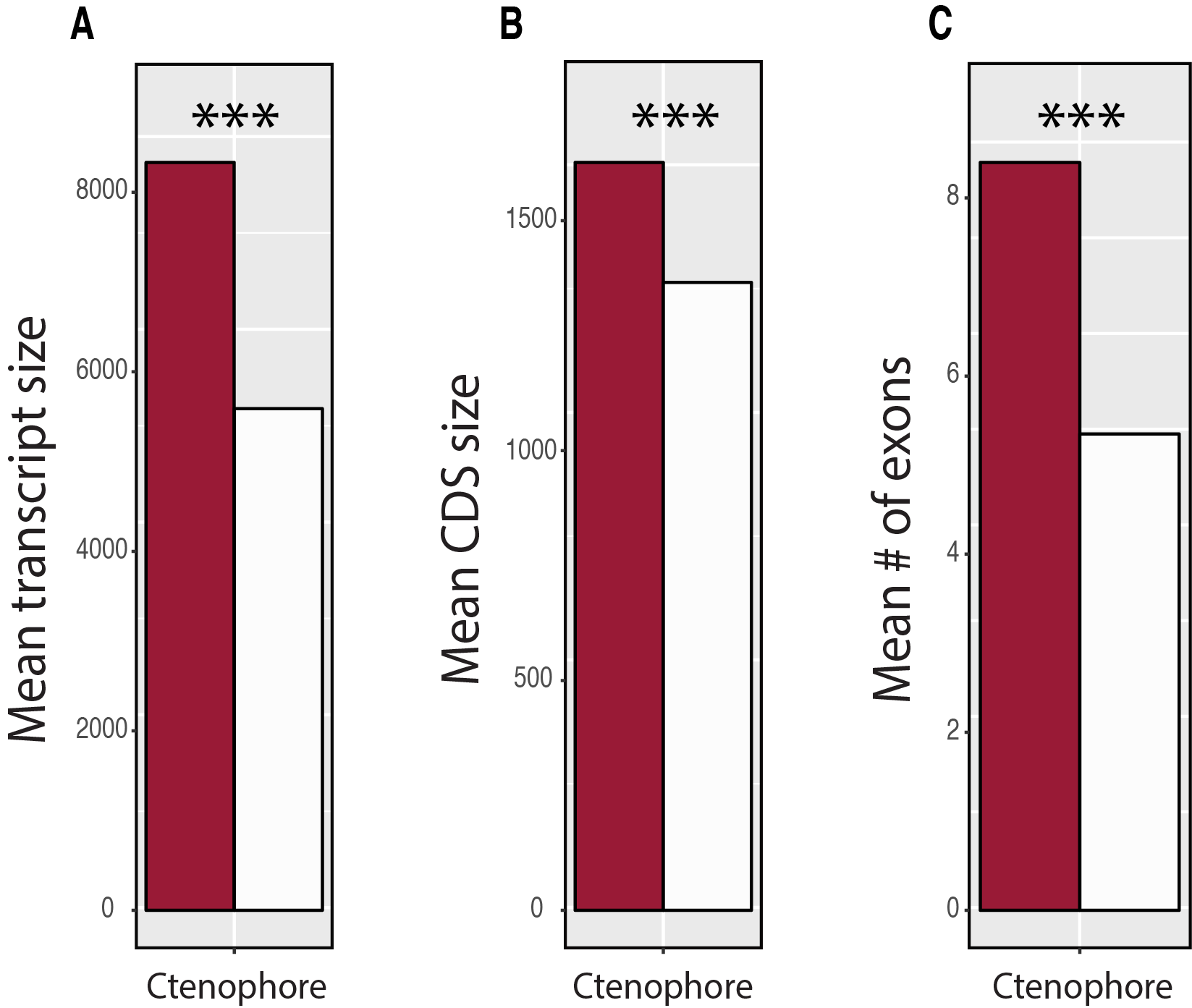

Supplement: Additional file 1: Figure S1. — Neural vs. non-neural gene length in ctenophores. (DOC 76 kb) [file 13062_2016_162_MOESM1_ESM.doc]
