# Supplementary material for: Neurogenomics and the role of a large mutational target on rapid behavioral change
Source: Biol Direct. 2016 Nov 8;11:60. doi: 10.1186/s13062-016-0162-1 (PMC5101817; doi:10.1186/s13062-016-0162-1)

Supplementary Figure 2. Frequency distributions of gene lengths for neurogenic genes, across multiple taxa.


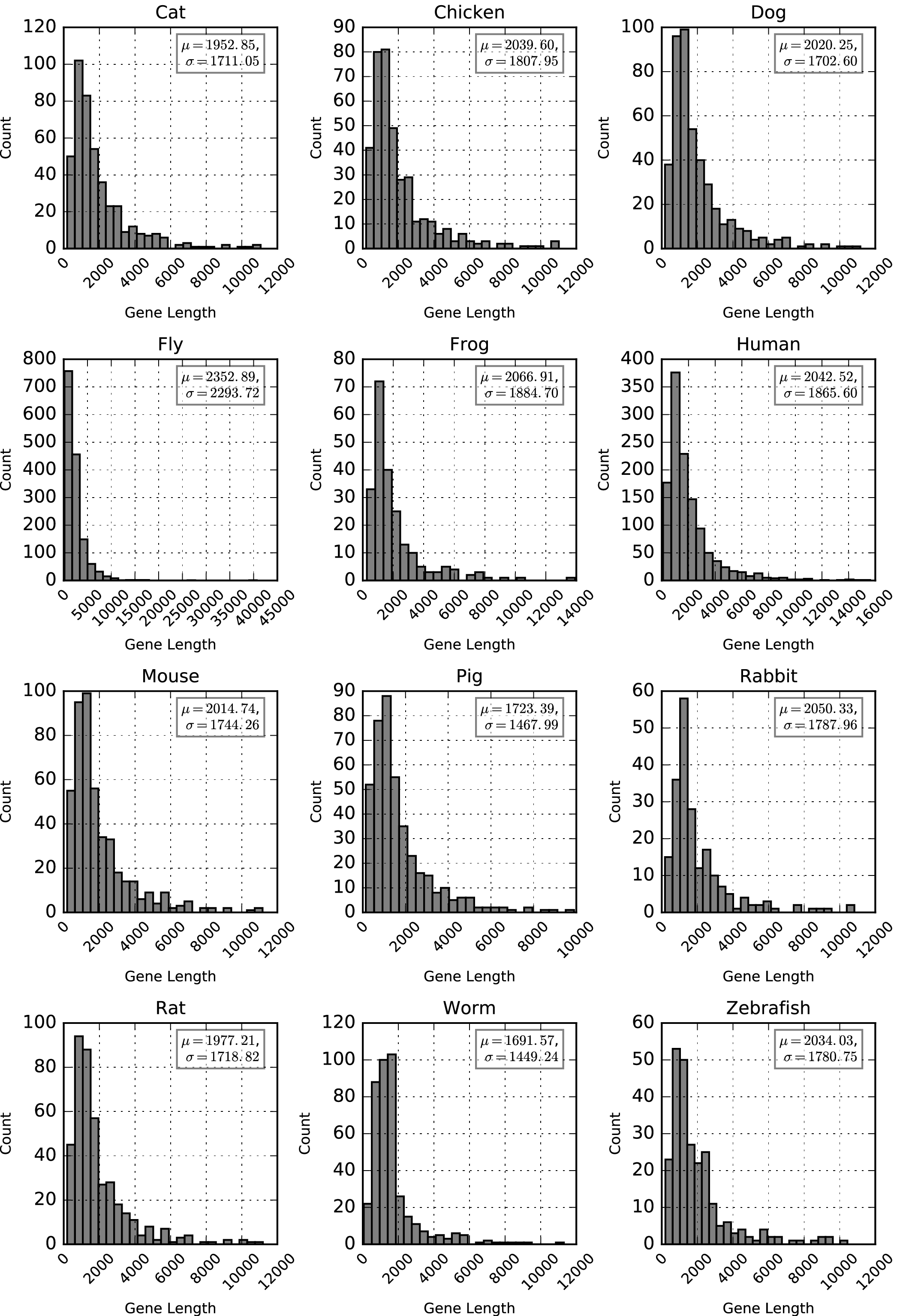

Supplement: Additional file 2: Figure S2. — Frequency distributions of gene lengths for neurogenic genes, across multiple taxa. (DOC 315 kb) [file 13062_2016_162_MOESM2_ESM.doc]
