# Supplementary material for: Neurogenomics and the role of a large mutational target on rapid behavioral change
Source: Biol Direct. 2016 Nov 8;11:60. doi: 10.1186/s13062-016-0162-1 (PMC5101817; doi:10.1186/s13062-016-0162-1)
Supplement: Additional file 3: Table S1. — Number of neurogenic genes identified across taxa. (DOC 28 kb) [file 13062_2016_162_MOESM3_ESM.doc]

Supplementary Table S1. Number of neurogenic genes identified across taxa.

| **Species** | **Number of neurogenic genes** |
| --- | --- |
| human | 1212 |
| rabbit | 208 |
| pig | 411 |
| dog | 443 |
| cat | 435 |
| mouse | 465 |
| rat | 419 |
| zebrafish | 248 |
| frog | 222 |
| worm  ctenophores | 408  848 |
| fly | 1486 |
